# Quality of life and stress level in adolescents with idiopathic scoliosis subjected to conservative treatment

**DOI:** 10.1186/1748-7161-8-S1-O61

**Published:** 2013-06-03

**Authors:** E Kinel, T Kotwicki, A Podolska, M Białek, W Stryła

**Affiliations:** 1 Department of Rehabilitation, University of Medical Sciences, Poznan, Poland; 2 Department of Paediatric Orthopaedics and Traumatology, University of Medical Sciences, Poznan, Poland; 3 FITS Center, Jawor, Poland

## Background

The quality of life and stress level of adolescents suffering from idiopathic scoliosis and submitted to brace treatment is the object of increasing interest of the professionals.

## Aim

The aim of the study was to evaluate the quality of life and stress level in adolescents with idiopathic scoliosis who are under brace treatment.

## Methods

It involved 45 adolescents, ages ranging between 10.0 and 15.0 years, all with Adolescent Idiopathic Scoliosis (AIS), with Cobb angle between 20-45 degrees. The adolescents were wearing the same kind of brace (Chêneau orthosis) for more than 3 months for at least 12h per day. Two questionnaires were used: the Brace Questionnaire (BrQ) and Bad Sobernheim Stress Questionnaire (BSSQ). The BrQ is an instrument for measuring quality of life of scoliotic adolescents who are being treated conservatively with wearing of a corrective brace. The BrQ consists of 34 Likert-scale items associated with eight domains. The BSSQ (Brace and Deformity) questionnaire estimates the stress scoliosis patients have whilst wearing their brace. The analysis considered the type of treatment, curve location, correlation of the total score with age, Cobb angle and Bunnell rotation angle.

## Results

The age was 13.6 ± 1.3 years. Cobb angle was 31.7 ± 7.6 degrees. The mean score for BrQ was 78.1 ± 11.3 points. Adolescents revealed higher score with BSSQ Deformity (median = 15) comparing to BSSQ Brace (median = 12).

## Conclusion

Conservative treatment does not severely impact on the quality of life of scoliotic adolescents. The adolescents who were under brace treatment suffered moderate level of stress from the deformity.
